# Multi-omics identification of immune-related biomarkers predicting tofacitinib response in rheumatoid arthritis

**DOI:** 10.3389/fimmu.2025.1703209

**Published:** 2026-01-26

**Authors:** Fangyi Lu, Yanshu Shao, Qilin Chen, Qi Liu, Huaxiang Liu, Zhen Liu, Yunfeng Li

**Affiliations:** 1 Department of Nephrology, Qilu Hospital, Shandong University, Jinan, Shandong, China; 2 Department of Rheumatology, Qilu Hospital, Shandong University, Jinan, Shandong, China; 3 Shandong Provincial Hospital Affiliated to Shandong First Medical University, Jinan, Shandong, China; 4 Department of Anatomy and Neurobiology, School of Basic Medical Sciences, Shandong University, Jinan, Shandong, China; 5 Department of Pediatric Surgery, Qilu Hospital, Shandong University, Jinan, Shandong, China

**Keywords:** immune-related biomarkers, multi-omics, precision medicine, rheumatoid arthritis, tofacitinib

## Abstract

**Background:**

Rheumatoid arthritis (RA) is a prototypical autoimmune disease characterized by chronic inflammation and immune dysregulation. Although Janus kinase (JAK) inhibitors such as tofacitinib have expanded therapeutic options, treatment responses remain heterogeneous and reliable predictors of efficacy are lacking.

**Methods:**

Peripheral blood mononuclear cells (PBMCs) and serum samples were collected from 14 patients with active RA before initiation of tofacitinib treatment. Patients were classified as responders or non-responders according to EULAR DAS28 criteria after treatment. An integrative multi-omics approach was applied, including RNA sequencing, miRNA sequencing, proteomics, and untargeted metabolomics. Comprehensive bioinformatics analyses were performed to identify potential candidate predictors of tofacitinib response. Key findings were further assessed through internal validation in an independent cohort of tofacitinib-treated RA patients and external validation using publicly available datasets.

**Results:**

Multi-omics analyses revealed upregulation of ribosomal proteins in PBMCs of responders, with *RPL21* emerging as a potential immune-related candidate. Consistently, hsa-miR-197-3p and hsa-miR-625-3p were downregulated in responders, suggesting possible regulatory roles in treatment efficacy. Proteomic profiling showed decreased serum apolipoproteins, particularly APOA1, while metabolomic analysis identified elevated choline, malate, and nervonic acid, reflecting immune-metabolic reprogramming. Integration of multi-omics data highlighted convergent immune pathways and identified exploratory candidate biomarkers associated with tofacitinib response.

**Conclusions:**

This study provides exploratory integrative multi-omics evidence linking immune-related transcriptomic, proteomic, and metabolic alterations to heterogeneous therapeutic responses in RA. The identified signatures improve our understanding of molecular pathways underlying JAK inhibition response and offer potential candidate biomarkers to guide personalized treatment strategies.

## Introduction

Rheumatoid arthritis (RA) is a chronic, systemic autoimmune disorder characterized by persistent synovial inflammation and progressive joint destruction, leading to pain, disability, and increased mortality (1). Despite substantial advancements in therapeutic options—including conventional synthetic disease-modifying antirheumatic drugs (csDMARDs), biologic DMARDs, and targeted small-molecule inhibitors such as Janus kinase (JAK) inhibitors—a significant proportion of patients fail to achieve adequate treatment response (2). Currently, therapeutic selection in RA remains largely empirical, relying on a trial-and-error strategy due to the lack of reliable predictive biomarkers.

Tofacitinib, the first approved oral JAK inhibitor for RA, targets the JAK/signal transducer and activator of transcription (STAT) signaling pathway, which plays a pivotal role in mediating inflammatory responses (3). Although tofacitinib has demonstrated clinical efficacy in many RA patients, considerable inter-individual variation in treatment response persists. This heterogeneity reflects the complex immunopathogenesis of RA and underscores the urgent need for a precision medicine approach to guide individualized therapy (4).

Emerging evidence suggests that integrative multi-omics approaches, combining transcriptomic, proteomic, and metabolomic data, can provide a more comprehensive understanding of disease biology and therapeutic response (5). Transcriptomics captures gene expression patterns on a genome-wide scale, offering insight into the transcriptional activity of immune and inflammatory pathways (6). In RA, transcriptomic profiling, especially through RNA sequencing (RNA-seq), has revealed distinct expression signatures associated with disease activity, immune cell composition, and therapeutic response (7–11). Proteomics offers insight into functional protein expression and post-translational modifications (12), while metabolomics reflects dynamic biochemical changes that mirror cellular states and immune activation (13). Despite their potential, multi-omics strategies remain underutilized in RA, particularly in the context of JAK inhibitor therapy.

**Figure 1:**
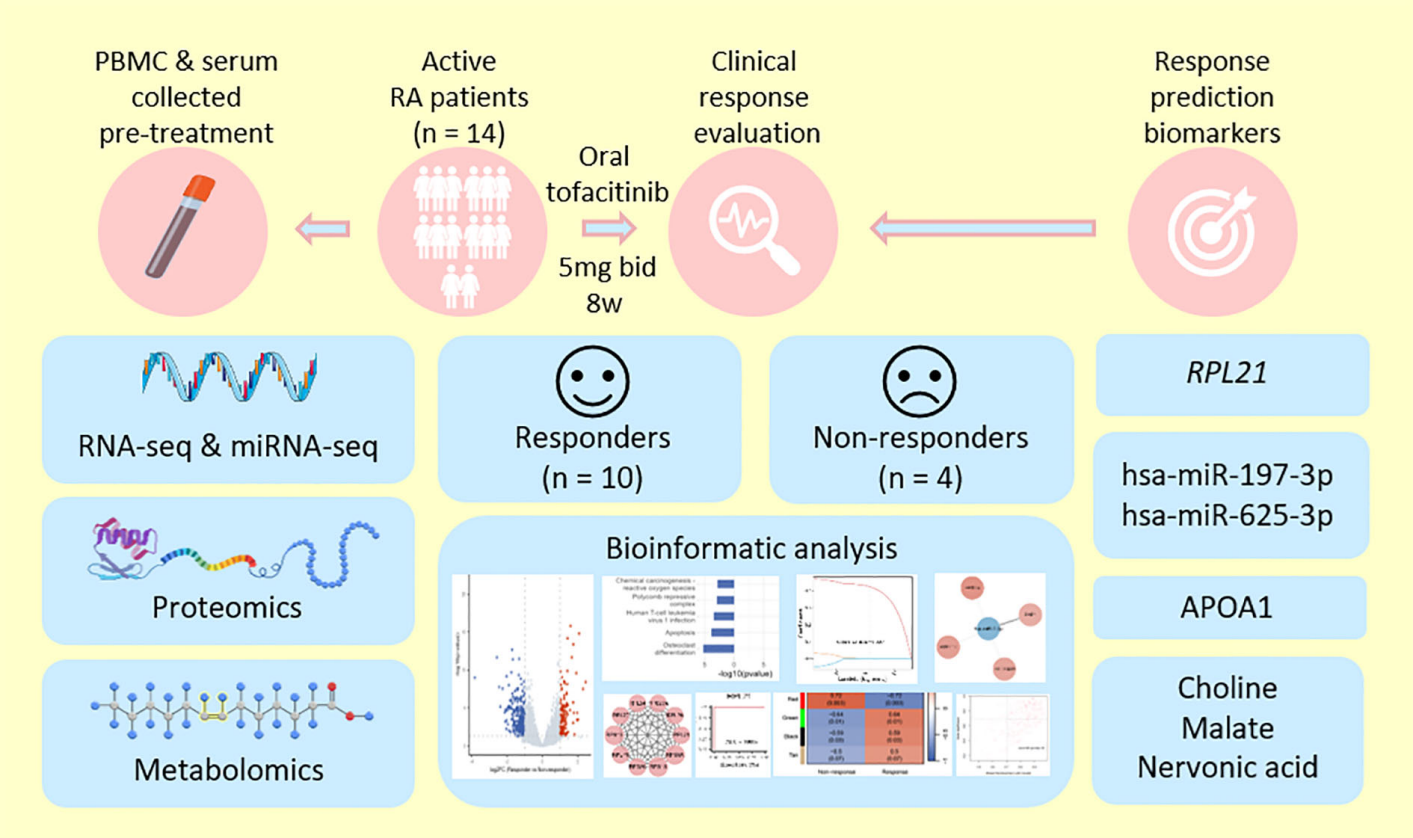
Graphical abstract illustrating the study design and main findings. Peripheral blood mononuclear cells (PBMCs) and serum samples were collected from 14 patients with active rheumatoid arthritis (RA) prior to tofacitinib treatment. Patients were stratified into responders (n = 10) and non-responders (n = 4) based on EULAR DAS28-defined clinical response. Comprehensive multi-omics profiling, including RNA sequencing (RNA-seq), miRNA-seq, proteomic, and metabolomic analyses, was performed. Differentially expressed mRNAs, miRNAs, proteins, and metabolites were identified and subjected to functional enrichment. Hub features were further prioritized using integrated network analyses and their predictive performance systematically evaluated. On this basis, key candidate biomarkers with potential clinical utility were proposed to facilitate individualized prediction of treatment response and to advance precision medicine strategies for RA.

To address this gap, our study aimed to systematically characterize the baseline molecular differences between RA patients with distinct responses to tofacitinib treatment using an integrative multi-omics framework. By analyzing transcriptomes of peripheral blood mononuclear cell (PBMCs), serum proteomes, and metabolomic profiles, we sought to identify predictive biomarkers associated with therapeutic efficacy (**Figure 1**). This comprehensive approach not only provides novel insights into the immunometabolic mechanisms underlying tofacitinib response but also contributes to the development of molecular tools to guide precision treatment strategies in RA. Ultimately, our findings may help overcome the limitations of conventional “one-size-fits-all” paradigms and support a more individualized approach to RA management.

## Materials and methods

### Clinical assessment

RA patients enrolled in this study were the same cohort described in our previous publication (14). Briefly, between April 2021 and December 2021, fourteen female patients with active RA were recruited and received oral tofacitinib (5 mg twice daily) for eight weeks. Inclusion criteria and management procedures have been detailed previously (14). In brief, all patients met the 2010 ACR/EULAR classification criteria for RA and had moderate-to-high disease activity (DAS28-ESR and DAS28-CRP more than 3.2). Eligible patients were non-smokers, had an inadequate response to csDMARDs, and had no prior exposure to JAK inhibitors or biological agents. Exclusion criteria included pregnancy, lactation, other autoimmune disorders, serious infections, malignancies, cardiovascular, cerebrovascular, or psychiatric diseases.

Response to tofacitinib was assessed based on changes in the DAS28 score: patients with DAS28-ESR and DAS28-CRP less than 3.2 and a decrease in DAS28 greater than 1.2 after treatment were classified as responders; others were considered non-responders. Detailed clinical outcomes have been reported previously (14) and are not re-analyzed here.

This study was approved by the Ethics Committee of Shandong Provincial Hospital (Approval No. 2021-126).

### Sample collection

Peripheral blood samples were collected from all enrolled RA patients at baseline, prior to initiation of tofacitinib treatment, as previously described (14). Whole blood was drawn into EDTA tubes for RNA extraction and into serum-separating tubes for proteomic and metabolomic analyses. PBMCs were isolated using Ficoll-Paque density gradient centrifugation within 2 hours of collection. Serum samples were obtained by centrifugation at 8,000 rpm for 8 minutes at 4°C and stored at –80°C until further use.

### RNA-seq

Total RNA was extracted from freshly isolated PBMCs using the TRIzol reagent (Invitrogen, USA) according to the manufacturer’s protocol. RNA integrity was assessed with a NanoDrop spectrophotometer (Thermo Scientific, USA). Deribosome strand-specific libraries were prepared using the NEB Next Ultra Directional RNA Library Prep Kit (NEB, USA) and quantified using the Agilent high sensitivity DNA assay on a Bioanalyzer 2100 system (Agilent, USA). Sequencing was performed on an Illumina NovaSeq 6000 platform (Illumina, USA). Each library yielded an average of **~**45 million paired-end reads, and > 90% of bases had Phred quality scores ≥ Q30. Raw reads were quality-filtered using Cutadapt to remove adapter sequences and low-quality reads (average quality < Q20). Only high-quality clean data were aligned to the human reference genome (GRCh38) using HISAT2. Differential gene expression analysis was conducted with DESeq2, which performs internal normalization based on size factors and estimates dispersion for each gene. Adjusted *P* values were computed using the Benjamini–Hochberg false discovery rate (FDR) method to control for multiple testing. Differentially expressed mRNAs (DEmRNAs) between responders and non-responders were identified based on a |log_2_ fold change (log_2_FC)| > 1 and *P* < 0.05.

Gene set enrichment analysis (GSEA) was first performed to explore global expression patterns and pathway enrichment among all genes. Subsequently, functional enrichment of DEmRNAs was performed using Gene Ontology (GO) and Kyoto Encyclopedia of Genes and Genomes (KEGG) pathway analyses. Protein–protein interaction (PPI) networks for DEmRNAs were constructed using the STRING database and visualized with Cytoscape software. Hub genes within the PPI network were identified using the CytoHubba plugin, and the top 10 genes were selected as candidate hub genes. Receiver operating characteristic (ROC) curve analysis was then performed to assess the predictive performance of these hub genes for treatment response.

To further investigate gene co-expression patterns, weighted gene co-expression network analysis (WGCNA) was performed to detect gene modules significantly associated with treatment response. Genes within the most relevant module that showed both gene significance (GS) and module membership (MM) values greater than 0.7 were intersected with the previously identified hub genes from the PPI analysis to derive robust candidate predictors. Finally, least absolute shrinkage and selection operator (LASSO) regression was applied to further refine and select key hub genes with optimal predictive performance.

### miRNA-seq

Total RNA extracted from PBMCs was also used for small RNA library construction using the NEB Next Multiplex Small RNA Library Prep Set for Illumina (NEB, USA) according to the manufacturer’s protocol. Sequencing was performed on an Illumina NovaSeq 6000 platform. Raw data were processed to remove adapters and low-quality reads, yielding an average of ~12 million reads per sample. Raw reads were processed by removing adaptor sequences and low-quality bases (average quality score < 20 within a 5 bp sliding window), and reads shorter than 18 nt were discarded. Clean reads of 18–36 nt were retained and identical sequences were collapsed to unique reads. Only samples with RNA integrity number > 7 were included in downstream analyses.

Clean reads were mapped to known miRNAs in miRBase. Differentially expressed miRNAs (DEmiRNAs) between responders and non-responders were identified using DESeq2, with normalization by size factors and multiple testing correction using the Benjamini–Hochberg FDR approach. MiRNAs with a |log_2_FC| > 1 and *P* < 0.05 were defined as significantly differentially expressed.

The ENCORI database was used to identify DEmiRNAs that potentially interact with the DEmRNAs. Spearman correlation analysis was then conducted on the predicted mRNA–miRNA interaction pairs to assess their expression correlations. Pairs showing a significant negative correlation (correlation coefficient (*r*) < –0.5 and *P* < 0.05) were selected for further analysis. These selected interactions were visualized as a network using Cytoscape. For the miRNAs within this network, ROC curve analysis was performed to evaluate their predictive performance for treatment response. miRNAs with an area under the curve (AUC) greater than 0.7 were further refined using LASSO regression to identify robust candidate biomarkers.

### Proteomics

Serum proteins were extracted and prepared according to standard data-independent acquisition (DIA) workflow protocols. High-abundance proteins were depleted and samples were processed and digested using filter-aided sample preparation with trypsin. Peptides were analyzed on a Q Exactive HF-X mass spectrometer (Thermo Scientific, USA) in DIA mode. Raw data were processed with Spectronaut and searched against the UniProt human database.

Differential expression analysis was performed using the limma package. Protein intensities were log_2_-transformed and normalized using the voom method. Linear models with empirical Bayes moderation were applied, and *P* values were adjusted for multiple testing using the Benjamini–Hochberg FDR method. Proteins with |log_2_FC| > 1 and *P* < 0.05 were considered differentially expressed. Functional enrichment, PPI network construction, hub protein selection, and ROC curve analysis were carried out following similar procedures described for the RNA-seq data. WGCNA was used to identify significant modules, and differential expressed proteins (DEPs) with GS and MM values > 0.7 in the top module were intersected with the PPI hub proteins to obtain robust candidate markers.

### Metabolomics

Serum samples were thawed on ice and pretreated with precooled methanol/acetonitrile/water (2:2:1, v/v) for protein precipitation, as described in the standard liquid chromatography–mass spectrometry protocol. After centrifugation and drying under vacuum, samples were redissolved, vortexed, and analyzed on an Agilent 1290 Infinity UHPLC coupled to a TripleTOF 6600 mass spectrometer (AB SCIEX, USA) in both positive and negative electrospray ionization modes. HILIC and RPLC separations were applied.

Raw data were converted to MzXML format using ProteoWizard and processed with XCMS for peak picking, retention time correction, and alignment. After initial processing, metabolites with >50% missing values within a group were removed, extreme values were filtered, and intensities were normalized by total peak area to ensure comparability across samples. Partial least squares discriminant analysis was performed to visualize group separation and obtain variable importance in projection (VIP) scores. A VIP score > 1 together with |log_2_FC| > 1 and *P* < 0.05, were used to identify significantly differential expressed metabolites (DEMs). KEGG pathway enrichment of differential metabolites was conducted using MetaboAnalyst 5.0. Spearman correlation analysis was performed to assess the association between differential metabolites and transcriptomic/proteomic features. Differential metabolites significantly correlated with multi-omics markers (|*r*| > 0.6, *P* < 0.05) were further filtered using LASSO regression to identify robust metabolic predictors.

### External transcriptomic validation using a public JAK inhibitor–treated cohort

To validate the transcriptional signatures identified in the discovery cohort, we analyzed the publicly available dataset GSE253495 from the Gene Expression Omnibus, which includes CD14^+^ monocytes from RA patients before and after 3 months of treatment with the JAK inhibitor upadacitinib (15). All patients demonstrated clinical improvement at follow-up. Differential gene expression between pre- and post-treatment samples was assessed with the limma package. Gene set enrichment was conducted using three complementary approaches: CAMERA, a competitive test that adjusts for inter-gene correlation; ROAST, a self-contained test providing a gene set-level significance estimate; and preranked GSEA, which evaluates enrichment based on the overall rank order of genes. This combination allows assessment of pathway activity from multiple statistical perspectives.

### Internal validation of baseline biomarkers in tofacitinib-treated RA patients

An independent internal validation cohort of RA patients treated with tofacitinib was established, including 10 responders and 4 non-responders (clinical characteristics are summarized in **Supplementary Table S6**). Baseline samples were collected prior to treatment initiation. Selected candidate biomarkers identified in the discovery analysis were assessed, including *RPL21*, hsa-miR-197-3p and hsa-miR-625-3p in PBMCs, both measured by quantitative PCR, serum APOA1 protein levels measured by immunoturbidimetric assay, and serum metabolites (choline, malate, and nervonic acid) quantified by mass spectrometry. Differences between responders and non-responders at baseline were evaluated using Student’s t-tests.

### Statistical analysis

All statistical analyses were performed using R software (version 4.2.3) unless otherwise specified. Detailed descriptions of the specific statistical methods, software packages, parameter settings, and thresholds used for each omics dataset are provided in the supplementary methods.

## Results

### Ribosomal proteins were upregulated in PBMCs of tofacitinib-responsive RA patients

**Figure 2:**
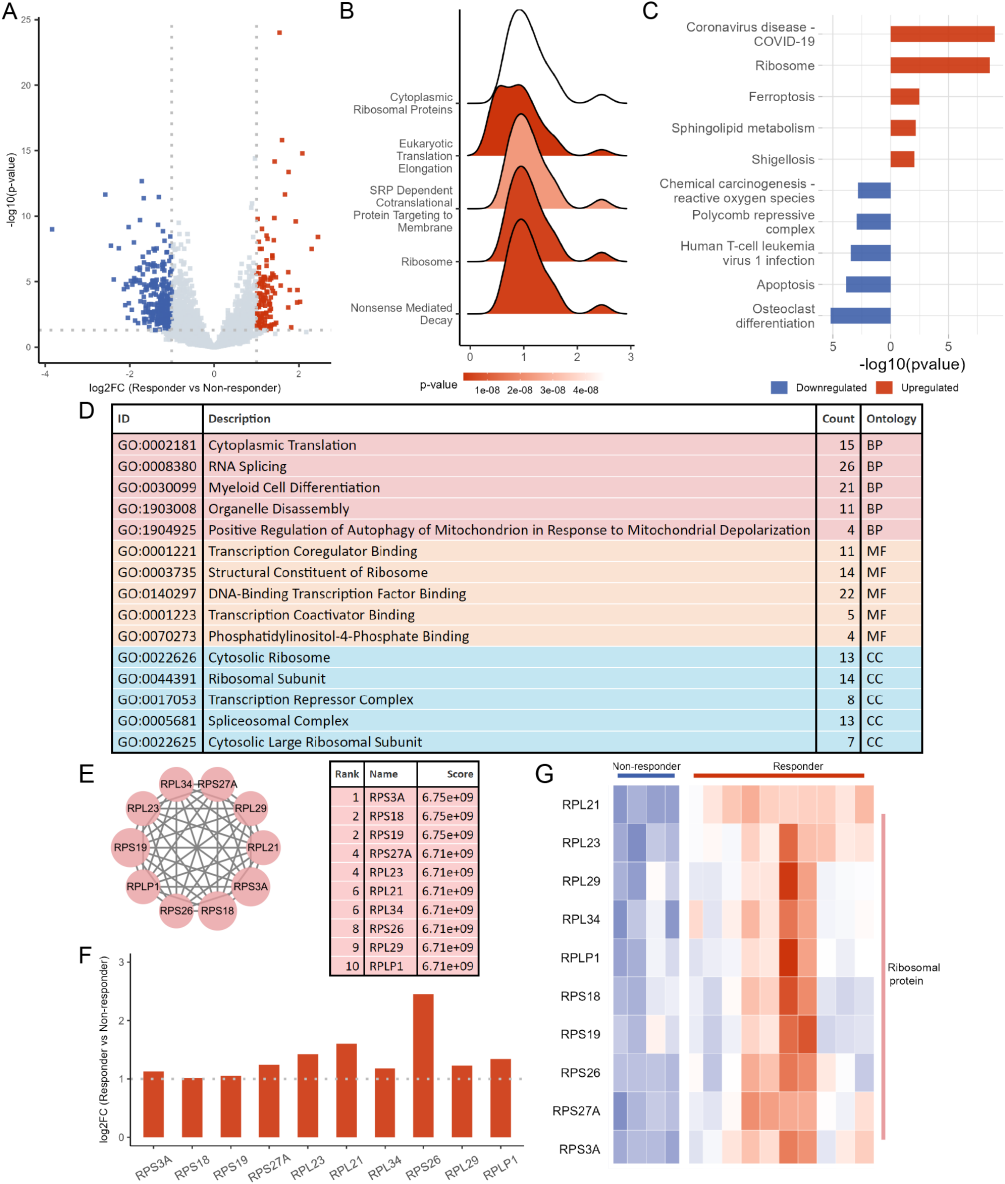
Identification of hub genes related to tofacitinib response in PBMCs from RA patients. **(A)** Volcano plot showing differentially expressed mRNAs (DEmRNAs) between responders (n = 10) and non-responders (n = 4). **(B)** Gene set enrichment analysis (GSEA) presenting the top 5 significantly enriched pathways. **(C)** KEGG pathway enrichment analysis of DEmRNAs, with upregulated and downregulated genes analyzed separately; the top 5 pathways for each are displayed. **(D)** Gene Ontology (GO) enrichment analysis of DEmRNAs, showing the top 5 terms for biological process (BP), cellular component (CC), and molecular function (MF). **(E)** Protein–protein interaction (PPI) network constructed from DEmRNAs, with the top 10 hub genes ranked by CytoHubba. **(F)** Bar plot illustrating the log_2_ fold change (log_2_FC) of the top 10 hub genes. **(G)** Heatmap displaying the expression patterns of the top 10 hub genes across samples.

RNA-seq profiles of PBMCs from RA patients prior to tofacitinib treatment were analyzed, and DEmRNAs were identified based on statistical criteria (|log_2_FC| > 1 and *P* < 0.05) (**Figure 2A**, **Supplementary Table S1**). Compared to non-responders, responders exhibited upregulation of 118 genes and downregulation of 309 genes. Subsequently, GSEA, KEGG, and GO enrichment analyses were performed on the differential expression data. Notably, different enrichment methods consistently identified ribosome-related entries. In the top 5 gene sets enriched by GSEA, both cytoplasmic ribosomal proteins and ribosome showed an upregulation trend (**Figure 2B**, **Supplementary Table S1**). The KEGG analysis of upregulated and downregulated DEmRNAs also highlighted the ribosome pathway among the top 5 enriched pathways (**Figure 2C**, **Supplementary Table S1**). Additionally, the top 5 terms in the GO analysis for biological process (BP), cellular component (CC), and molecular function (MF) included terms such as cytoplasmic translation, structural constituent of ribosome, and cytosolic ribosome (**Figure 2D**, **Supplementary Table S1**).

We further constructed a PPI network of DEmRNAs using the STRING database. The top 10 hub genes within the network were identified using the cytoHubba plugin in Cytoscape software, which included *RPS3A*, *RPS18*, *RPS19*, *RPS27A*, *RPL23*, *RPL21*, *RPL34*, *RPS26*, *RPL29*, and *RPLP1* (**Figure 2E**). Interestingly, all of them were RPs and upregulated in PBMCs of tofacitinib-responsive RA patients compared to non-responders (**Figures 2F, G**).

### RPL21 in PBMCs of RA patients exhibited potential as a predictive biomarker for tofacitinib response

**Figure 3:**
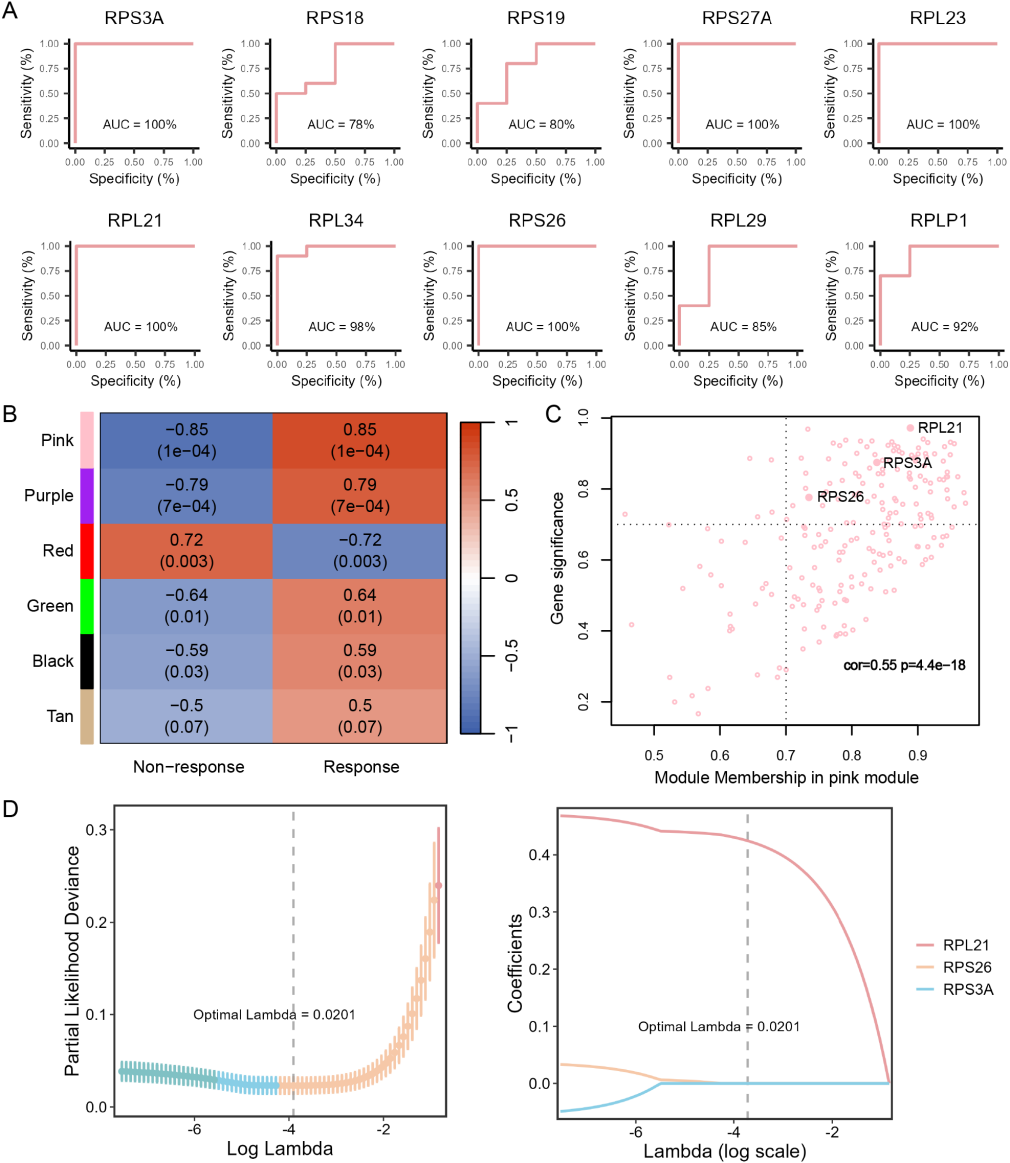
Screening hub genes predictive of tofacitinib response in RA. **(A)** Receiver operating characteristic (ROC) curves illustrating the predictive performance of the top 10 hub genes for distinguishing responders from non-responders. **(B)** Heatmap showing the correlations between co-expression network modules and treatment response; the six modules with the highest absolute correlation coefficients are displayed. **(C)** Scatter plot depicting the relationship between gene significance (GS) and module membership (MM) within the pink module, which showed the strongest association with tofacitinib response. **(D)** Least absolute shrinkage and selection operator (LASSO) regression plots, including the cross-validation error curve and the coefficient profiles, were used to identify hub genes with optimal predictive value for tofacitinib response.

ROC analysis of the 10 hub genes identified from the PPI network using cytoHubba was performed to assess their accuracy in distinguishing between tofacitinib responders and non-responders in RA patients (**Figure 3A**, **Supplementary Table S1**). Among these, *RPS3A*, *RPS27A*, *RPL23*, *RPL21*, and *RPS26* demonstrated the highest predictive accuracy. Additionally, a weighted gene co-expression network was constructed using transcriptomic data from PBMCs of RA patients prior to tofacitinib treatment, with a soft thresholding power of β = 8 to ensure approximate scale-free topology. Thirteen modules were identified in the network, with each module consisting of genes exhibiting similar expression patterns. We correlated module eigengenes with clinical traits, identifying the pink module, which was found to be most strongly associated with tofacitinib response (**Figure 3B**). Genes within the pink module, including *RPL21*, *RPS3A*, and *RPS26*, were identified as key drivers of the response to tofacitinib, based on their high intramodular connectivity and correlation with treatment outcomes (**Figure 3C**, **Supplementary Table S1**). Furthermore, LASSO regression was conducted for *RPL21*, *RPS3A*, and *RPS26* to identify key biomarkers predictive of tofacitinib response in RA patients. The optimal λ value was selected via 10-fold cross-validation, resulting in the identification of significant gene *RPL21*. Our findings suggest that *RPL21* could serve as a potential biomarker for predicting tofacitinib treatment response and guiding personalized therapy in RA patients (**Figure 3D**).

### hsa-miR-197-3p and hsa-miR-625-3p in PBMCs of RA patients as promising predictors of response to tofacitinib

**Figure 4:**
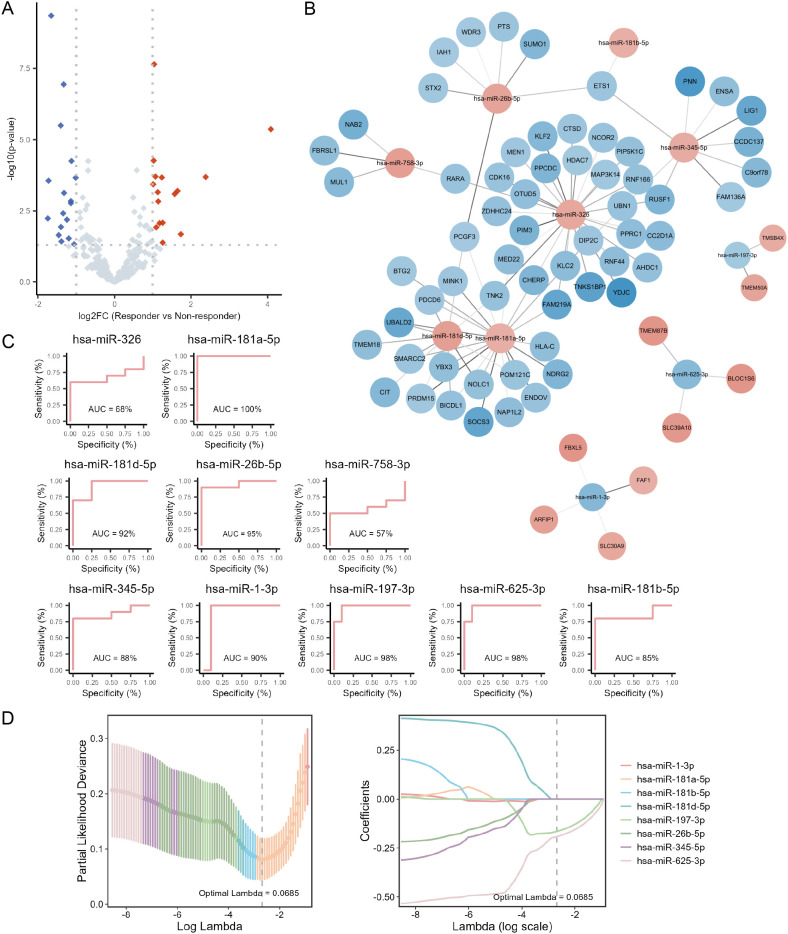
Network-driven discovery of miRNAs predictive of tofacitinib response in RA patient PBMCs. **(A)** Volcano plot showing differentially expressed miRNAs (DEmiRNAs) between responders (n = 10) and non-responders (n = 4). **(B)** mRNA–miRNA interaction network constructed from DEmiRNAs and DEmRNAs. The color intensity of the connecting lines indicates the absolute value of the Spearman correlation coefficient between mRNA–miRNA pairs; the color depth of the nodes represents the absolute value of log_2_FC, with red indicating upregulation and blue indicating downregulation. **(C)** ROC curves for the miRNAs identified from the network, evaluating their predictive performance for tofacitinib response. **(D)** LASSO regression plots, including the cross-validation curve and coefficient profile, used to further select robust predictive miRNAs.

Differential expression analysis was conducted to identify DEmiRNAs. In comparison to non-responders, sixteen miRNAs were found to be upregulated, while 17 miRNAs showed downregulation in PBMCs from RA patients responsive to tofacitinib (**Figure 4A**, **Supplementary Table S2**). We predicted the interactions between these DEmiRNAs and their target DEmRNAs using the ENCORI database. Subsequently, the Spearman correlation for the predicted miRNA-mRNA pairs were assessed, and those with a correlation coefficient of *r* < -0.5 and *P* < 0.05 were selected and visualized in the ceRNA network (**Figure 4B**, **Supplementary Table S2**). ROC analysis was performed on the DEmiRNAs within the network, and those with an AUC > 0.7, suggesting a higher predictive accuracy for tofacitinib responsiveness, underwent further LASSO analysis (**Figure 4C**, **Supplementary Table S2**). This analysis pinpointed hsa-miR-197-3p and hsa-miR-625-3p as potential biomarkers (**Figure 4D**).

### Apolipoproteins were downregulated in serum of tofacitinib-responsive RA patients

**Figure 5:**
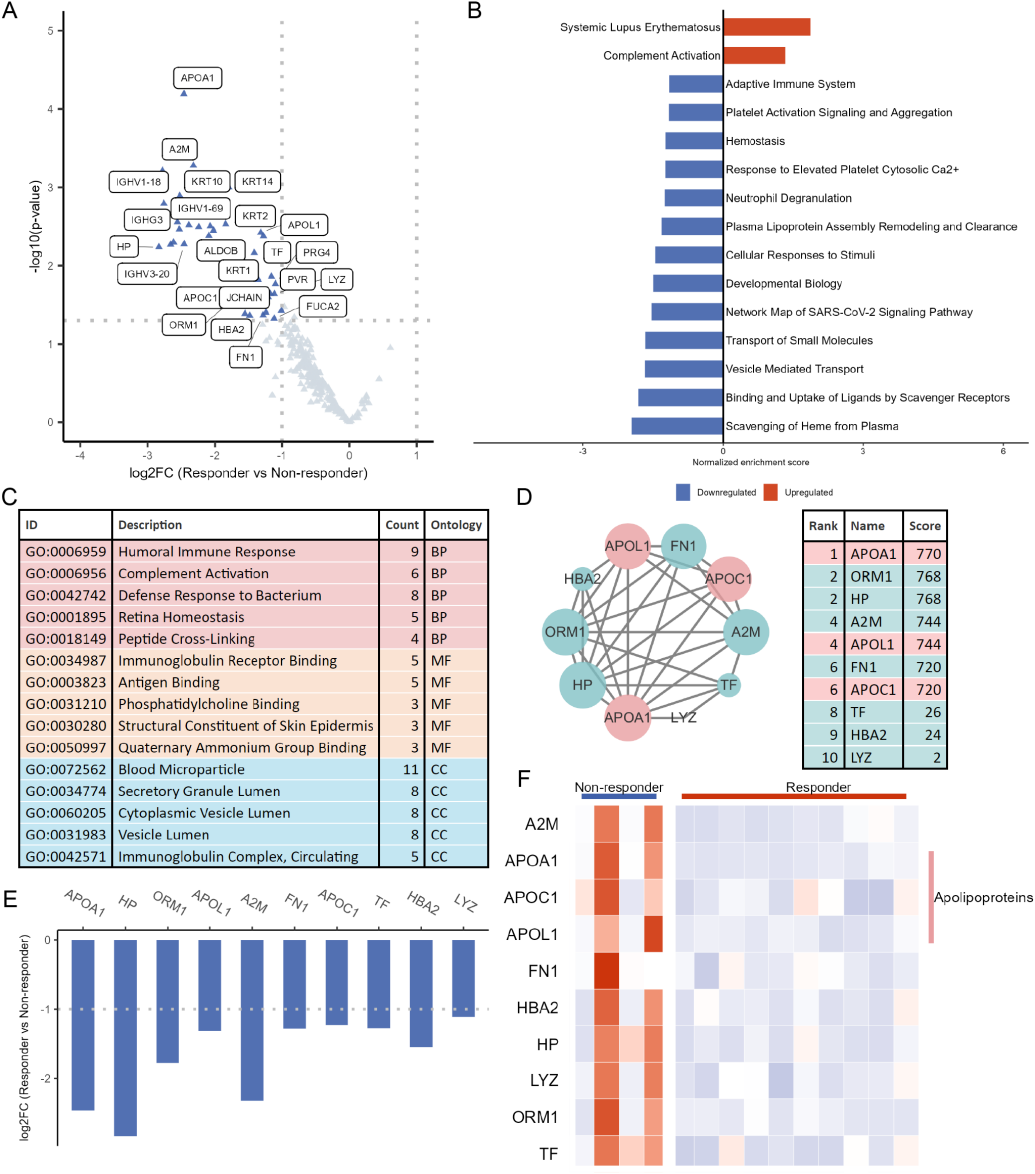
Identification of hub proteins related to tofacitinib response in serum from RA patients. **(A)** Volcano plot showing differentially expressed proteins (DEPs) between responders (n = 10) and non-responders (n = 4). **(B)** GSEA presenting the top 15 significantly enriched pathways. **(C)** GO enrichment analysis of DEPs, presenting the top 5 terms for BP, CC, and MF. **(D)** PPI network constructed from DEPs, with the top 10 hub proteins identified by CytoHubba. **(E)** Bar plot illustrating the log_2_FC of the top 10 hub proteins. **(F)** Heatmap displaying the expression profiles of the top 10 hub proteins across samples.

Through differential expression analysis of the serum proteome in RA patients before tofacitinib treatment, a total of 43 DEPs were identified, all of which were downregulated in tofacitinib responders in comparison to non-responders (**Figure 5A**, **Supplementary Table S3**). Subsequent enrichment analyses were performed. GSEA revealed pathways related to complement activation, adaptive immune system, and plasma lipoprotein assembly remodeling and clearance (**Figure 5B**, **Supplementary Table S3**). GO enrichment identified key terms such as humoral immune response, complement activation, and immunoglobulin receptor binding (**Figure 5C**, **Supplementary Table S3).** A PPI network was constructed, which highlighted several hub proteins, including APOA1, ORM1, HP, A2M, APOL1, FN1, APOC1, TF, HBA2, and LYZ. Among these, APOA1, APOL1, and APOC1 are apolipoproteins (**Figure 5D**). **Figures 5E and 5F** illustrated the expression patterns of these hub proteins, with reduced levels of apolipoproteins observed in the serum of RA patients who responded to tofacitinib compared to non-responders.

### Serum APOA1 levels in RA patients demonstrated potential as a biomarker for predicting response to tofacitinib treatment

**Figure 6:**
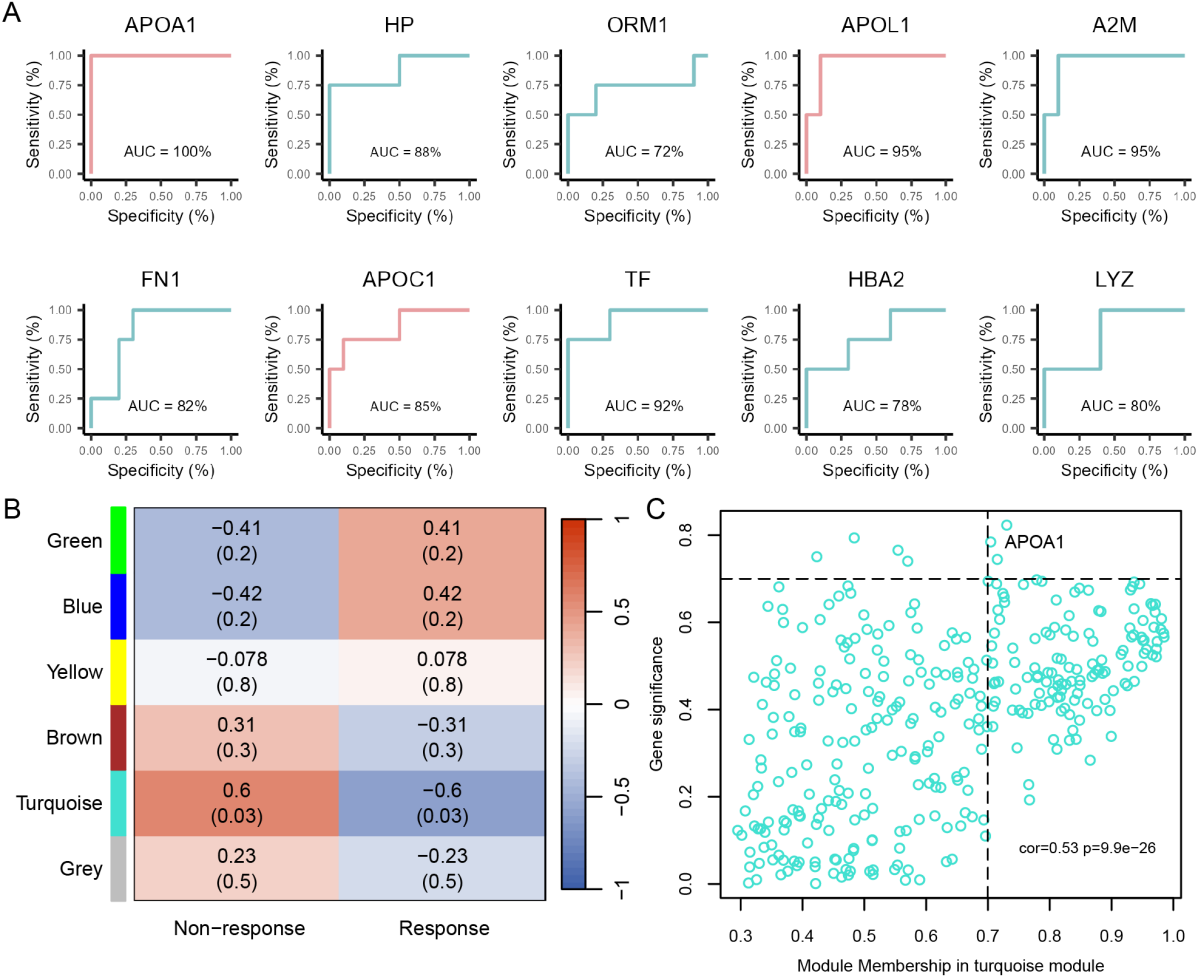
Screening hub proteins predictive of tofacitinib response in RA. **(A)** ROC curves illustrating the predictive performance of the top 10 hub proteins for distinguishing responders from non-responders. **(B)** Heatmap showing the correlations between co-expression network modules and treatment response. **(C)** Scatter plot depicting the relationship between GS and MM within the turquoise module, which showed the strongest association with tofacitinib response.

We performed ROC analysis on the key proteins identified from the PPI network to assess their accuracy in predicting RA patients’ response to tofacitinib. Among them, APOA1 exhibited the highest predictive performance (**Figure 6A**, **Supplementary Table S3**). Next, a scale-free co-expression network was constructed based on proteomic data, with the soft threshold set to β = 3, successfully identifying 6 distinct modules (**Figure 6B**). Among these, the turquoise module showed the strongest correlation with tofacitinib treatment response. Core regulatory factors within this module were selected based on high gene significance and module membership, and their intersection with hub proteins from the PPI network was analyzed (**Supplementary Table S3**). Notably, APOA1 was identified as a central node in both networks, suggesting its potential as a predictive biomarker for tofacitinib response in RA patients (**Figure 6C**). These findings provide new insights into precision medicine for RA.

### Serum metabolites predictive of tofacitinib response in RA patients

**Figure 7:**
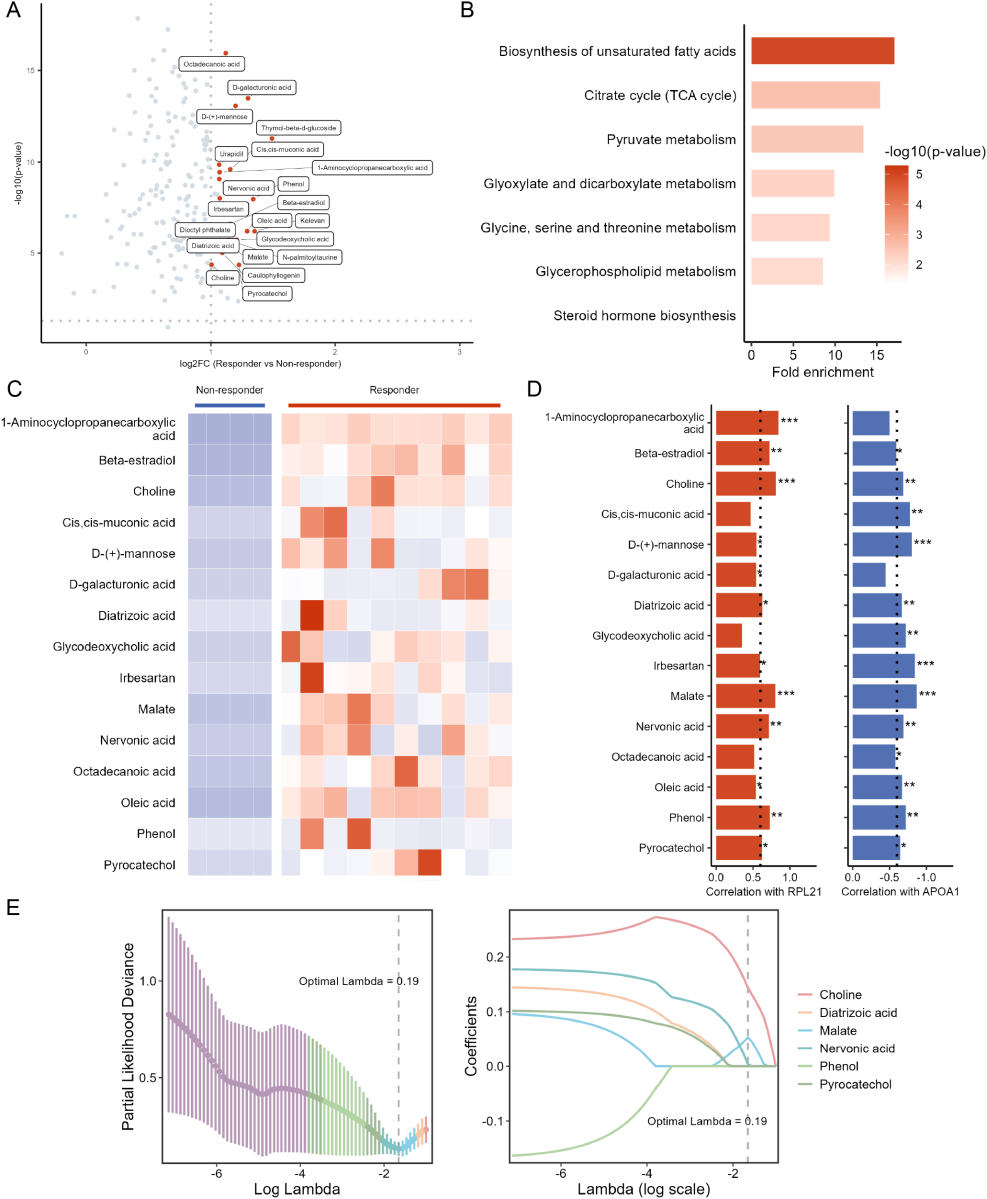
Serum metabolomic analysis of RA patients to identify metabolites predictive of tofacitinib response. **(A)** Volcano plot showing differentially expressed metabolites (DEMs) between responders (n = 10) and non-responders (n = 4). **(B)** KEGG pathway enrichment analysis of the DEMs. **(C)** Heatmap displaying the expression patterns of the DEMs across samples. **(D)** Correlation analysis between selected DEMs and key hub features identified from transcriptomic (*RPL21*) and proteomic (APOA1) analyses, presented as bar plots of Spearman correlation coefficients. Asterisks denote correlation significance (*P < 0.05, **P < 0.01, ***P < 0.001). **(E)** LASSO regression model for selecting robust serum metabolites with predictive value for tofacitinib response.

RA patients were stratified based on their response to tofacitinib treatment, and differential expression analysis was performed on their pre-treatment serum metabolomics data. The analysis identified 15 significantly upregulated differential metabolites between responders and non-responders (**Figures 7A, B**; **Supplementary Table S4**). KEGG pathway enrichment revealed that these metabolites were primarily involved in biosynthesis of unsaturated fatty acids, citrate cycle, and pyruvate metabolism (**Figure 7C**). Further Spearman correlation analysis was conducted to assess the relationship between these metabolites and known predictive biomarkers (*RPL21* and APOA1), identifying 6 metabolites that exhibited strong correlations (|*r*| > 0.6, *P* < 0.05) with both *RPL21* and APOA1 (**Figure 7D**). Finally, LASSO regression was applied for feature selection, and 3 serum metabolites, choline, malate, and nervonic acid, were determined to have significant predictive value of tofacitinib response (**Figure 7E**).

### Consistent association of ribosome-related transcriptional programs with JAK inhibitor treatment

**Figure 8:**
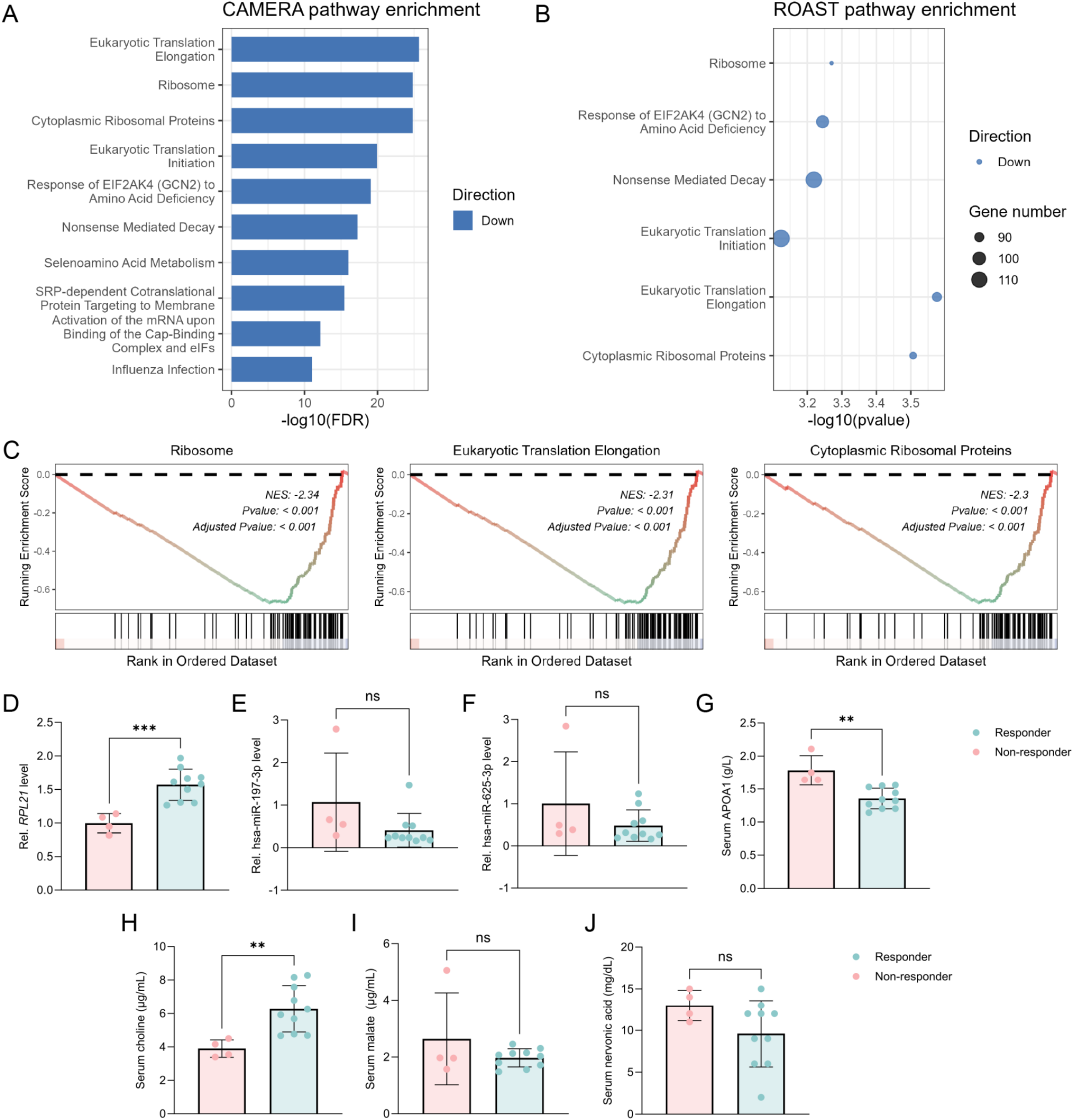
Integrated pathway-level external validation and internal validation of baseline biomarkers associated with tofacitinib response. **(A)** Bar plot showing the top 10 pathways identified by CAMERA analysis in the external validation transcriptomic dataset (post-treatment vs pre-treatment), ranked by false discovery rate (FDR). **(B)** Dot plot of pathways identified by ROAST analysis that were significantly enriched (*P* < 0.05) and overlapped with those detected by CAMERA. **(C)** GSEA plots for three representative pathways that were consistently identified by CAMERA, ROAST, and GSEA in the external validation dataset and were also observed in the tofacitinib discovery cohort. **(D)** Baseline expression of *RPL21* in PBMCs. **(E)** Baseline expression of hsa-miR-197-3p in PBMCs. **(F)** Baseline expression of hsa-miR-625-3p in PBMCs. **(G)** Baseline serum APOA1 protein levels. **(H)** Baseline serum choline levels. **(I)** Baseline serum malate levels. **(J)** Baseline serum nervonic acid levels. Statistical significance between tofacitinib responders and non-responders was assessed using Student’s t-tests. Asterisks denote correlation significance (**P < 0.01, ***P < 0.001).

To externally validate our findings, we analyzed the publicly available transcriptomic dataset GSE253495, which profiled CD14^+^ monocytes isolated from RA patients before and after a 3-month period of treatment with the JAK inhibitor upadacitinib. All patients demonstrated clinical improvement at follow-up. Differential expression analysis identified few significantly altered individual genes (**Supplementary Table S5**), prompting pathway-level analyses using CAMERA, ROAST, and GSEA. Notably, all three methods consistently revealed significant enrichment of ribosome-related pathways, including ribosome, cytoplasmic ribosomal proteins and eukaryotic translation elongation (**Figures 8A–C**, **Supplementary Table S5**). These pathways showed a coordinated downregulation following upadacitinib treatment. Importantly, the same ribosome-associated pathways were significantly upregulated at baseline in the tofacitinib-responsive group of our primary cohort. The consistent pattern of baseline upregulation in treatment-responsive patients followed by suppression after effective JAK inhibition across independent datasets supports the potential value of ribosome-related transcriptional programs as predictive markers of therapeutic response in RA.

### Consistent baseline multi-omic differences between tofacitinib responders and non-responders

In an independent internal validation cohort of tofacitinib-treated RA patients, baseline levels of selected multi-omic biomarkers were assessed. *RPL21* in PBMCs, serum APOA1, and choline levels were significantly different between responders and non-responders (**Figures 8D, G, H**), with consistent directionality compared to the discovery cohort, whereas other candidates showed no significant differences (**Figures 8E, F, I, J**).

## Discussion

Although tofacitinib has been extensively studied in RA and has demonstrated significant clinical efficacy and a favorable safety profile, particularly in patients with inadequate response to prior treatments, it remains ineffective in a subset of patients due to genetic heterogeneity and microenvironmental differences (16). Treatment failure not only increases healthcare costs but also may delay the optimal therapeutic window, thereby elevating the risk of joint erosion progression (17). Given this clinical challenge, the identification of biomarkers to guide personalized tofacitinib therapy has emerged as an important research direction. In this exploratory study, we employed a multi-omics analysis of peripheral blood, integrating whole-transcriptome sequencing of PBMCs with serum proteomic and metabolomic profiling. We identified several candidate biomarkers, including *RPL21*, hsa-miR-197-3p, hsa-miR-625-3p, APOA1, choline, malate, and nervonic acid, which showed differential expression between tofacitinib responders and non-responders. These findings provide hypothesis-generating insights into potential molecular signatures associated with treatment outcomes, though they are not definitive conclusions.

At the transcriptomic level, we observed a prominent enrichment of ribosome-related pathways in responders. This finding was not limited to the discovery cohort but was independently validated in an external dataset. While ribosomes are conserved in structure, recent studies have revealed heterogeneity across tissues and diseases, implying possible regulatory roles beyond protein synthesis (18). In RA, several RPs such as RPL7, RPS19, and RPL23A have been implicated as autoantigens contributing to disease pathogenesis (19–21). Among ribosomal genes, *RPL21* emerged as a central candidate, showing higher baseline expression in responders and significant upregulation confirmed by quantitative PCR in an independent internal validation cohort. The *RPL21* gene encodes ribosomal protein L21, a component of the 60S ribosomal subunit. Although the role of RPL21 in RA remains unclear, previous studies have linked RPL21 to various immune-related diseases, including immunodeficiency (22), Alzheimer’s disease (23), and cancers (24, 25). Nevertheless, this observation represents an association rather than a causal mechanism, and further studies are required to establish its biological role.

MiRNAs are increasingly recognized as potential indicators of treatment response in RA. Previous work has linked miRNAs such as miR-132-3p, miR-146a-5p, and miR-155-5p to methotrexate resistance (26), while others, including miR-27a-3p, miR-22, and miR-886-3p, have been associated with adalimumab outcomes (27). In our cohort, higher pre-treatment expression of hsa-miR-197-3p and hsa-miR-625-3p correlated with better response to tofacitinib. These miRNAs have not been previously reported in RA but are implicated in various cancers (28, 29). However, these miRNAs did not demonstrate statistically significant changes in subsequent validation analyses. This discrepancy may reflect limited sample size, biological variability, or context-dependent regulation. Therefore, these miRNAs should be regarded as exploratory signals requiring further investigation, rather than validated biomarkers at this stage.

Serum proteomic profiling in RA may reveal mechanisms of disease pathogenesis and aid in management. Several autoantibodies and immune cell–related markers have shown potential for predicting treatment response (30). In proteomic profiling, we observed lower baseline serum levels of apolipoproteins (APOA1, APOC1, and APOL1) in responders, with APOA1 showing the strongest predictive value. Importantly, APOA1 downregulation was confirmed in internal validation using an independent clinical assay, reinforcing its reproducibility. As components of high-density lipoprotein (HDL), these apolipoproteins are closely linked to lipid dysregulation in RA (31). Dyslipidemia, particularly reduced HDL cholesterol (HDL-c), occurs in up to half of RA patients, with APOA1 levels declining in parallel with HDL-c and inversely correlating with inflammatory burden (31, 32). Given its role in HDL metabolism and inflammation, this observation is biologically plausible. However, whether APOA1 directly influences treatment efficacy remains uncertain. Metabolomics is a powerful approach for early diagnosis and treatment monitoring in RA. Altered metabolic pathways influence inflammatory mediator release and contribute to joint degeneration and muscle wasting. Identifying specific metabolites may improve the accuracy of RA diagnosis and prognosis (33). In this study, among the identified metabolites, serum choline levels were significantly elevated in responders and were validated in an independent cohort, whereas malate and nervonic acid did not show consistent changes upon validation. Choline is phosphorylated by choline kinase α (ChoKα) in inflammatory macrophages in RA, facilitating phospholipid synthesis and cytokine release (34). Tumor necrosis factor (TNF) and platelet-derived growth factor (PDGF) further enhance ChoKα activity in fibroblast-like synoviocytes (FLS), exacerbating FLS activation and joint destruction (35). Malate, a key intermediate of the tricarboxylic acid cycle, links glycolysis to mitochondrial metabolism. Inhibition of malate dehydrogenase reduces inflammatory cytokines in RA (36). Nervonic acid, though not directly linked to RA, has shown pro-inflammatory effects in other inflammatory contexts (37). Elevated choline levels may therefore indicate heightened immune-metabolic activity that is particularly susceptible to JAK pathway inhibition. In contrast, malate and nervonic acid should be interpreted as hypothesis-generating metabolic signals, warranting further investigation in larger cohorts.

Several limitations of this study merit consideration. First, despite validation efforts, the overall sample size remains modest, and all participants were female, limiting generalizability. Second, although external validation supported ribosome-related transcriptomic signatures, comprehensive multi-omics validation across independent cohorts remains challenging due to data availability. Nonetheless, by integrating discovery, external validation, and internal experimental confirmation, our study provides a strengthened evidentiary framework compared with purely exploratory omics analyses.

In conclusion, through an integrative multi-omics approach complemented by external and internal validation, we identified ribosome-associated transcriptomic programs, *RPL21* upregulation, APOA1 downregulation, and elevated serum choline as reproducible molecular features associated with favorable response to tofacitinib in RA. These findings provide exploratory insights into the interplay between immune regulation and shaping therapeutic outcomes. While preliminary, this work lays the groundwork for future studies aiming to validate these markers in larger, multi-center cohorts and to advance precision medicine strategies for RA.

## Data Availability

The RNA sequencing data generated in this study have been deposited in the ArrayExpress repository under accession number E-MTAB-16533. Due to ethical restrictions and the lack of consent from some participants for public data sharing, raw sequencing data from a subset of patients could not be made publicly available. The publicly available dataset includes sequencing data from 10 patients.
