# Supplementary material for: Multi-omics identification of immune-related biomarkers predicting tofacitinib response in rheumatoid arthritis
Source: Front Immunol. 2026 Jan 26;16:1703209. doi: 10.3389/fimmu.2025.1703209 (PMC12884169; doi:10.3389/fimmu.2025.1703209)
Supplement: Supplementary file 1 [file Table1.docx]

**Supplementary methods**

*Power analysis*

A post hoc power analysis was conducted to evaluate the adequacy of the sample size for the exploratory multi-omics analyses. Given the limited availability of well-characterized responder and non-responder samples and the exploratory nature of this study, the analysis was based on a large effect size, which has been reported in responder-non-responder comparisons in transcriptomic, proteomic, and metabolomic studies. Assuming a two-sided significance level of α = 0.05, a sample size of 10 responders and 4 non-responders yielded an estimated statistical power exceeding 80%. These results support that the current sample size is sufficient to detect biologically meaningful differences between groups and is appropriate for hypothesis-generating multi-omics analyses.

*Differential expression analysis*

Differentially expressed mRNAs (DEmRNAs) and differentially expressed miRNAs (DEmiRNAs) between responders and non-responders were identified using the DESeq2 package (version 1.38.3). Prior to differential expression analysis, low-expressed mRNAs/miRNAs were filtered out by retaining only those with read counts greater than 1 in all samples. The DESeq function with default parameters was applied, which internally estimates size factors for normalization, calculates dispersion, and performs Wald tests for differential expression. Adjusted *P* values were computed using the Benjamini–Hochberg false discovery rate (FDR) method to control for multiple testing. Those mRNAs/miRNAs with |log₂ fold change (log₂FC)| > 1 and *P* < 0.05 were considered significantly differentially expressed.

Differentially expressed proteins (DEPs) were identified using the limma package (version 3.54.2). Protein expression data were log_2_-transformed and normalized using the voom method. Linear models were fitted with group as the main variable, and empirical Bayes moderation was applied to improve variance estimation. *P* values were adjusted for multiple testing using the Benjamini–Hochberg FDR method. Proteins with |log₂FC| > 1 and *P* < 0.05 were considered as DEPs.

Differentially expressed metabolites (DEMs) were identified based on the following criteria: variable importance in projection (VIP > 1.0) obtained from the partial least squares discriminant analysis model constructed using the ropls package (version 1.30.0) with 7-fold cross-validation and 200 permutation tests, paired Student’s t-test with *P* < 0.05, and absolute log₂FC (|log₂FC| > 1) relative to the baseline group.

*Functional enrichment and pathway analysis*

Gene set enrichment analysis (GSEA) was performed on both RNA-seq and proteomic datasets, as well as on the publicly available transcriptomic dataset GSE253495, using the clusterProfiler package (version 4.15.1) in R. Both mRNAs and proteins were ranked according to log₂FC between groups. Enrichment was assessed against the canonical pathways in curated gene sets form the Molecular Signatures Database. The GSEA function with default parameters was applied, with multiple testing correction performed using the Benjamini–Hochberg FDR method.

DEmRNAs and DEPs were subjected to functional enrichment analysis using the clusterProfiler package. Over-representation analysis was performed using enrichGO function for Gene Ontology (GO). Analyses covered the biological process (BP), cellular component (CC), and molecular function (MF) categories. Multiple testing was controlled by FDR.

DEmRNAs were split into upregulated (log₂FC > 1 and *P* < 0.05) and downregulated (log₂FC < −1 and *P* < 0.05) groups. Kyoto Encyclopedia of Genes and Genomes (KEGG) pathway over-representation analysis was performed separately for the upregulated and downregulated gene sets using the enrichKEGG function from the clusterProfiler package. Multiple testing was controlled by FDR.

KEGG pathway enrichment analysis of differential expressed metabolites (DEMs) was conducted using the Enrichment Analysis module in MetaboAnalyst (version 5.0). Over-representation analysis was performed by submitting the HMDB IDs of DEMs, with the KEGG database selected as the reference library, and the results were visualized as bar plots.

*Protein–protein interaction (PPI) network construction*

PPI networks were constructed for DEmRNAs and DEPs using the STRING database (version 12.0) with a confidence score > 0.4. The resulting interaction networks were imported into Cytoscape (version 3.10.3) for visualization and further topological analysis. Hub genes and proteins were identified using the CytoHubba plugin in Cytoscape, with nodes ranked according to the Maximal Clique Centrality (MCC) algorithm. The top 10 nodes with the highest MCC scores were defined as hub candidates. To visualize their differential expression, log₂FC of these hub genes/proteins was plotted as bar graphs using the ggplot2 package (version 3.4.2) in R, and their expression patterns across samples were further illustrated by heatmaps.

*Correlation analysis*

Spearman correlation coefficients between variables were calculated using the cor function in R. Statistical significance of the correlations was determined by computing *P* values with the corPvalueStudent function from the WGCNA package (version 1.72-1). Correlations with *P* < 0.05 were considered statistically significant.

*Receiver operating characteristic (ROC) curve analysis*

ROC curve analysis was performed to evaluate the diagnostic performance of hub genes, hub proteins, and selected miRNAs using the roc function from the pROC package (version 1.18.0) in R. For each molecule, the area under the curve (AUC) was calculated to assess its predictive ability, and the 95% confidence interval (CI) for the AUC was estimated using DeLong’s method, which provides a nonparametric approach for comparing correlated ROC curves.

*Weighted gene co-expression network analysis (WGCNA)*

WGCNA was performed separately on RNA-seq and proteomic datasets using the WGCNA package in R to identify co-expression modules and explore their relationships with tofacitinib response. Initially, expression matrices were filtered to remove low-expression features, and the data were normalized. Outlier samples were identified by hierarchical clustering and removed. Pairwise Spearman correlations between genes/proteins were computed to construct a weighted co-expression network. The soft-thresholding power (β) was selected using the pickSoftThreshold function to achieve scale-free topology (R² > 0.8). Subsequently, gene/protein modules were identified using the blockwiseModules function, which constructs an adjacency matrix with the selected β, transforms it into a topological overlap matrix (TOM) to assess network interconnectedness, and then applies hierarchical clustering combined with dynamic tree cutting, with the minimum module size set to 30. Module eigengenes (MEs), representing the first principal component of each module, were correlated with experimental traits using Spearman correlation, and statistical significance was assessed with the corPvalueStudent function from WGCNA. Modules showing significant correlations were selected for further analysis. For each module, gene significance (GS) was defined as the Spearman correlation between gene expression and the trait of interest, while module membership (MM) was calculated as the correlation between gene expression and the corresponding ME. Hub genes or proteins within significant modules were defined based on high MM and GS.

*Least absolute shrinkage and selection operator (LASSO) regression analysis*

To further identify key predictive biomarkers, we performed LASSO regression using the glmnet package (v4.1-8) in R. The glmnet function was first applied to fit a penalized regression model across a series of penalty parameters (λ) along the regularization path. To mitigate overfitting, 10-fold cross-validation was applied during model training using the cv.glmnet function, which returns the value of λ that minimizes the cross-validation error (lambda.min). Candidate features with non-zero coefficients at the optimal λ were retained as potential predictors for treatment response.

*Pathway-level gene set analysis using CAMERA and ROAST*

The publicly available transcriptomic dataset GSE253495 provided as FPKM were imported into R for analysis. Expression values were log₂-transformed and normalized using the limma package. Gene set testing was performed using CAMERA and ROAST, two complementary gene set analysis methods implemented in limma. Gene sets were obtained from canonical pathways in curated gene sets form the Molecular Signatures Database. CAMERA was applied as a competitive gene set test that accounts for inter-gene correlation, while ROAST was used as a self-contained gene set test based on rotation-based resampling (999 rotations). For both methods, pathway-level significance was evaluated by comparing post-treatment versus pre-treatment samples. Pathways with a FDR < 0.05 for CAMERA or *P* < 0.05 for ROAST were considered statistically significant.

*RNA extraction and quantitative PCR for mRNA*

Total RNA was extracted using an RNA extraction kit according to the manufacturer’s instructions (DP431, Tiangen, China). Reverse transcription of mRNA was performed using the FastKing RT Kit (KR116, Tiangen, China). Quantitative PCR was conducted using SYBR Green chemistry (GK8020, GENEray, China) on CFX384 real-time PCR system (BioRad, USA). The thermal cycling conditions were as follows: initial denaturation at 95°C for 10 min; 40 cycles of denaturation at 95°C for 10 s and annealing/extension at 60°C for 34 s, followed by melt curve analysis to confirm amplification specificity. Primer sequences used for quantitative PCR are listed in Supplementary Table S6. Relative mRNA expression levels were calculated using the 2^−ΔΔCt^ method.

*MiRNA extraction and quantitative PCR*

Total RNA was extracted using the same protocol described above. Reverse transcription of miRNAs was performed using stem-loop primers with the FastKing RT Kit (KR116, Tiangen, China). Quantitative PCR for miRNAs was performed using TaqMan probe-based chemistry with the AceQ^TM^ qPCR Probe Master Mix (Q112-02, Vazyme, China). PCR amplification was performed on CFX384 platform (BioRad, USA) with the following conditions: initial denaturation at 95°C for 10 min, followed by 40 cycles of 95°C for 10 s and 60°C for 34 s. Primer sequences used for quantitative PCR are listed in Supplementary Table S6. Relative miRNA expression levels were calculated using the 2^−ΔΔCt^ method.

*Immunoturbidimetric measurement of serum APOA1*

Serum apolipoprotein A-1 (APOA1) levels were measured using an immunoturbidimetric assay with the Tina-quant Apolipoprotein A-1 ver.2 reagent (Roche Diagnostics, Germany). Measurements were performed on an automated clinical chemistry analyzer according to the manufacturer’s instructions. Serum samples were processed under standardized conditions, and quality control procedures were applied throughout the assay to ensure analytical reliability.

*Quantification of serum choline by targeted ultra-performance liquid chromatography-mass spectrometry (UPLC-MS/MS)*

Serum choline levels were quantified using a targeted UPLC–MS/MS approach. Serum samples were extracted with methanol/acetonitrile (1:1, v/v), followed by vortexing for 60 s and low-temperature ultrasonication for 30 min. After protein precipitation at -20°C for 1 h, samples were centrifuged at 12,000 rpm for 10 min at 4°C. The supernatant was collected, vacuum-dried, and reconstituted in 100 μL of 30% acetonitrile. Following a second centrifugation at 12,000 rpm for 10 min at 4°C, the supernatant was subjected to UPLC–MS/MS analysis. Chromatographic separation was performed on a Vanquish UPLC system (Thermo Scientific, USA) using an Atlantis™ Premier BEH Z-HILIC column (2.5 μm). MS detection was carried out on a Q Exactive™ Hybrid Quadrupole-Orbitrap™ mass spectrometer (Thermo Scientific, USA) equipped with a heated electrospray ionization source operating in positive ion mode with selected ion monitoring. Choline was identified based on retention time and accurate mass matching, and absolute quantification was achieved using external calibration curves generated from authentic standards. Quality control samples were analyzed at regular intervals to monitor instrument stability, with a relative standard deviation < 30% considered acceptable.

*Quantification of serum malate by targeted UPLC–MS/MS*

Serum malate levels were quantified using a targeted UPLC–MS/MS method following chemical derivatization. Serum samples were mixed with extraction solvent (methanol/chloroform, 7:3, v/v) and incubated on ice for 30 min. After the addition of water, samples were centrifuged at 12,000 rpm for 10 min at 4°C, and the supernatant was collected. The extraction procedure was repeated twice, and the supernatants were combined. Aliquots of extracted samples were subjected to derivatization using 3-nitrophenylhydrazine and 1-(3-dimethylaminopropyl)-3-ethylcarbodiimide at 40°C for 30 min.

Chromatographic separation was performed on a Vanquish UPLC system (Thermo Scientific, USA) equipped with a Waters BEH C18 column (50 × 2.1 mm, 1.8 μm). Mass spectrometric analysis was carried out on a Q Exactive™ Hybrid Quadrupole-Orbitrap™ mass spectrometer (Thermo Scientific, USA) operating in negative ion mode using Full MS/ddMS² acquisition. Malate was identified based on retention time and accurate mass, and absolute quantification was performed using external standard calibration curves. Quality control samples were analyzed at regular intervals to monitor instrument stability, with a relative standard deviation < 30% considered acceptable.

*Quantification of serum nervonic acid by gas chromatography–mass spectrometry (GC–MS)*

Nervonic acid levels were quantified as part of a 37-fatty-acid profiling analysis using GC–MS. Briefly, an appropriate amount of sample was weighed into a 100 mL colorimetric tube and hydrolyzed with 2 mL of 95% ethanol and 4 mL of water. After thorough mixing, 10 mL of 8.3 mol/L hydrochloric acid was added, and the mixture was incubated in an 80°C water bath for 40 min with intermittent shaking every 10 min. The hydrolysate was then cooled to room temperature. Lipids were extracted by adding 10 mL of 95% ethanol, followed by three sequential extractions with a total of 100 mL of a petroleum ether/diethyl ether mixture. The combined organic phases were evaporated to dryness to obtain total lipids. The lipid extract was subjected to saponification with 4 mL of 2% sodium hydroxide in methanol at 45°C for 20 min, followed by methylation using 4 mL of 14% boron trifluoride–methanol solution at 45°C for an additional 20 min. After cooling to room temperature, fatty acid methyl esters were extracted with 3 mL of n-hexane, vortexed for 2 min, and allowed to separate. The upper organic phase was collected, filtered through a 0.45 μm membrane, and analyzed by GC-MS, which was performed on a Trace 1310 gas chromatograph coupled to an ISQ mass spectrometer (Thermo Scientific, USA) equipped with a TG-FAME capillary column (50 m × 0.25 mm × 0.20 μm). The MS was operated in electron ionization mode. Nervonic acid was identified based on retention time matching with authentic standards and characteristic mass spectra. Quantification was performed using external calibration curves, and the content of nervonic acid was calculated according to standard fatty acid conversion formulas.
